# A hierarchical method based on active shape models and directed Hough transform for segmentation of noisy biomedical images; application in segmentation of pelvic X-ray images

**DOI:** 10.1186/1472-6947-9-S1-S2

**Published:** 2009-11-03

**Authors:** Rebecca Smith, Kayvan Najarian, Kevin Ward

**Affiliations:** 1 Dept. Computer Science, Virginia Commonwealth University, 401 W. Main St., Richmond, VA, USA; 2 Dept. Emergency Medicine, Virginia Commonwealth University, Richmond, VA, USA

## Abstract

**Background:**

Traumatic pelvic injuries are often associated with severe, life-threatening hemorrhage, and immediate medical treatment is therefore vital. However, patient prognosis depends heavily on the type, location and severity of the bone fracture, and the complexity of the pelvic structure presents diagnostic challenges. Automated fracture detection from initial patient X-ray images can assist physicians in rapid diagnosis and treatment, and a first and crucial step of such a method is to segment key bone structures within the pelvis; these structures can then be analyzed for specific fracture characteristics. Active Shape Model has been applied for this task in other bone structures but requires manual initialization by the user. This paper describes a algorithm for automatic initialization and segmentation of key pelvic structures - the iliac crests, pelvic ring, left and right pubis and femurs - using a hierarchical approach that combines directed Hough transform and Active Shape Models.

**Results:**

Performance of the automated algorithm is compared with results obtained via manual initialization. An error measures is calculated based on the shapes detected with each method and the gold standard shapes. ANOVA results on these error measures show that the automated algorithm performs at least as well as the manual method. Visual inspection by two radiologists and one trauma surgeon also indicates generally accurate performance.

**Conclusion:**

The hierarchical algorithm described in this paper automatically detects and segments key structures from pelvic X-rays. Unlike various other x-ray segmentation methods, it does not require manual initialization or input. Moreover, it handles the inconsistencies between x-ray images in a clinical environment and performs successfully in the presence of fracture. This method and the segmentation results provide a valuable base for future work in fracture detection.

## Background

Prompt and appropriate treatment of pelvic injury is vital to patient survival. Pelvic fractures are among the most life-threatening injuries that can be suffered by a major trauma patient. They are strongly associated with impact injuries, particularly moving vehicle accidents. One six-year case study of 119 male pelvic trauma patients at a large level 1 trauma center found that 42.8% of fractures were caused by motor vehicle collisions (MVC) [1]. Other mechanisms causing pelvic disruption include falls from a height (30%) and crush injury under heavy weights (10%) [2]. Fractures can also cause laceration of the surrounding soft tissue and neural and vascular structures, and involve neighboring structures such as the urogenital system, leading to very complex injuries and a high mortality rate. Pelvic injuries caused by high-energy impacts that destroy the integrity of the pelvic ring are associated with a mortality rate of between 5 and 20% [2], and many of those who survive suffer permanent disability. Acetabular fractures, although not as potentially life threatening, are also associated with significant morbidity [3].

In polytraumatized patients, the ATLS Guidelines recommed pelvic x-ray imaging as a vital first diagnostic step [4]. The process is fast, cheap, and causes relatively little disturbance to the injured patient. The resulting x-ray image can quickly reveal the extent of damage to the bone structure of the pelvis, such as fracture, pelvic ring disruption, and widening of the pubic bone gap. However, the structure of the pelvis is complex, and fractures may be hard to recognize on low resolution x-rays; discussion with physicians suggested uncertainty even among medical professionals. This suggests that a system capable of quickly identifying pelvic fracture would prove valuable in a trauma center environment. Since fracture location has considerable impact on both severity and treatment of the injury, as well as the appearance of the fracture in a radiograph image, the first step in constructing such a system is to correctly segment the pelvis into distinct regions. This paper focuses on detection of the left and right iliac crests, the pelvic ring, the left and right femurs and the left and right pubis. By isolating and separating these three structures within x-ray images, we build a valuable base for future automatic fracture identification. More importantly, our algorithm is entirely automated, unlike other x-ray segmentation methods which require the user to manually initialization detection for each structure.

### Prior work

Multiple previous studies have focused on the segmentation of MRI and CT images, including those of the pelvic and abdominal areas [5,6]. However, compared to other radiological imaging modalities, segmentation of x-rays has not been as widely researched. This may be due to the additional complexities involved in radiograph imaging. Different tissues may have similar absorption rates, leading to blurred edges and a lack of detail [7]. Delineating bone matter from soft tissue can also be challenging due to low contrast, particularly when multiple bones overlap. This is especially problematic in pelvic radiographs, as the femurs overlap with the main structure of the pelvis [8]. Pelvic x-rays are prone to another specific complication: the prescence of gas inside the colon, which causes dark shadows to appear over the iliac fossa. [9]

One early attempt at radiograph segmentation by Manos [10] employed region growing and merging according to size, similarity, and connectivity. The generated regions were then labeled according to their grey level information. This approach was limited, however, as it considered neither spatial information nor existing knowledge of anatomy. Other early studies considered the problem of identifying lung regions in chest radiographs. Vittitoe [11] applied Markov random field models, while Duryea [12] and Pietka [13] used rule-based heuristics. The same problem was addressed by McNitt-Gray [14], instead using a classifier approach; each pixel was placed into one of several anatomic classes according to various locally calculated features.

More recent efforts have focused on the use of deformable models, due to their ability to segment complex structures and account for real-world variability in shape and appearance. A learning-based model approach can incorporate prior knowledge of the problem, and learn the variation from a set of annotated training examples. Two specific learning-based algorithms, Active Shape Model (ASM) and Active Appearance Model (AAM), have proven successful in segmenting CT, MRI, and x-ray images [15-17]. Boukala [18] successfully applied the ASM algorithm to pelvic x-rays; however, individual structures were not segmented, and the focus was on pathological deformities in hip replacement patients, rather than pelvic fractures sustained in traumatic injury.

### Our approach

**Figure 1:**
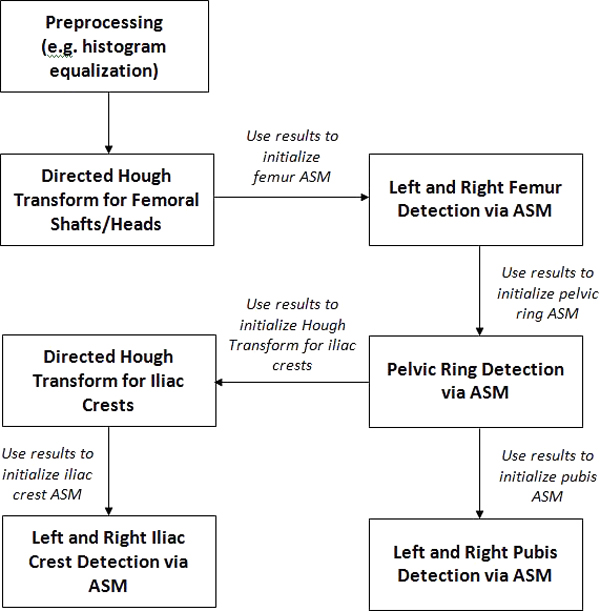
**Flowchart of pelvic segmentation process**. This flowchart illustrates the steps of the hierarchical initialization and segmentation algorithm.

**Figure 2:**
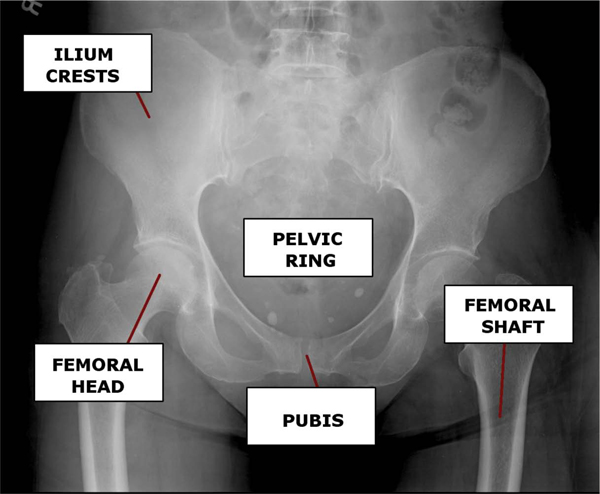
**Bones of the Pelvis**. An x-ray showing key structures of interest located within the human pelvis.

In a clinical environment, there are numerous issues complicating the segmentation of pelvic x-rays. The novelty of the system described in this paper is its ability to handle the following challenges: uncertain horizontal and vertical position of the patient, variations in patient pose angle, and inconsistencies due to differences in x-ray machine configuration. Unlike other segmentation algorithms, our method also performs successfully in the presence of fracture, as is necessary for clinical use. Our segmentation approach is hierarchical; the algorithm begins with identification of the femoral shafts, and uses the information obtained at each successive stage to initialize the next. The shafts of the femur are distinctly visible in the majority of pelvic x-rays, and are identified via directed Hough straight line detection. The knowledge of the shafts' width and position is used to direct the Hough transform to locate the circular femoral heads. Once the head and shaft positions are known, the ASM algorithm can be initialized and the combined femur structure is detected. Three specific reference points - the estimated pelvic ring center, and two points on the identified femur model shapes - are then used to initialize ASM for pelvic ring detection. This method may not not be accurate in the presence of very severe deformity, where a femoral head has been forced inside the pelvic ring. However, our algorithm may not be required for such obvious cases, where the physician can clearly observe the deformity in the x-ray image. Once the size and position of the pelvic ring is known, ASM can be initialized for detection of the left and right pubis via two reference points. Directed Hough Transform is also used to locate the iliac crests based on the known position of the pelvic ring. The entire process is outlined in Figure 1. Figure 2 presents an overview of the pelvic structure.

## Methods

### Data

The data was provided by Carolinas Healthcare System (CHS), and consisted of 20 pelvic fracture patients and 52 x-ray images (pre- and post-surgery). The full dataset, including post-operative patients, was used to calculate a probability distribution for femoral shaft and head size, as described later in the paper. The dataset was then filtered to include only those x-rays taken upon the patient's initial arrival at the hospital, prior to surgery and internal fixation, to be used for training the segmentation algorithm. This set contained 25 images. All x-rays were resized to a standard width of 1000 pixels, though image height varied across the set. Due to the significant differences in pelvic anatomy between male and female patients, we chose to focus on male patients. The user of the final system would therefore specify the patient's gender before segmentation can be performed; however, this information is readily available and should not complicate the process in a trauma center environment. The training set consisted of 5 patients (7 images), and the test set contained 15 patients (18 images). We anticpated that our algorithm can handle female patients using simple changes to initialization that account for the anatomical differences.

### Detection of femoral shafts

The femurs are among the most distinct structures in a pelvic x-ray, and typically suffer little deformity except in cases of severe femoral fracture. Therefore, the left and right femoral shafts are used as reference points to determine the patient's horizontal displacement from the center. These shafts form clear straight lines within the image, and so initial detection can be performed using directed Hough transform that is restricted to ± 45 degrees of the vertical. This restriction avoids horizontal artifacts towards the base of the x-ray, such as measurement marks. The Hough Transform is a feature extraction method best suited to detecting regular geometric shapes with clear parametric forms. The straight lines of the femoral shafts can be described by the following parametric equation:

(1)

where *ρ *is the normal distance from the origin to the line, and *θ *is the angle between the normal and the x-axis.

Hough transform is dependent on the quality of initial edge detection, and may therefore prove problematic in noisy or complex images such as radiographs. To circumvent this, the algorithm initially applies histogram equalization and an unsharp mask to the original pelvic x-rays, to increase the definition of the femoral shaft edges. This is followed by rough Canny edge detection. The Hough transform is applied only to the lowest 10% of the edge-detected image, which handles the difference in shaft length caused by the lack of a standard vertical position for patients during x-ray imaging. The transform is also restricted to lines that are ± 45 degrees either side of the vertical, to prevent the detection of horizontal line artifacts at the base of the x-ray, such as measurement bars. Once a candidate set of lines is generated, the algorithm then calculates which line pairs most likely correspond to the shaft edges. Following the method developed by Chen et. al [15], lines are paired according to width between them and their intensity gradient direction. Femoral shaft width varies from patient to patient, due to differences in build. However, examination of our training samples found that the probability of two lines forming a shaft contour could be estimated based on the distance between them. To increase the sample size, we used the full dataset, which also contained x-rays taken after the installation of non-femur hardware (which did not affect raw shaft width). We found that the shaft width has a unimodal distribution, which can be modelled by a Gaussian *G*_*w*_, with *μ*_*w *_= 87.75 pixels and *σ*_*w *_= 5.0817 pixels. The probability *p*_*i *_that the pair of lines *i *in the test image form a shaft contour, based on width alone, is given by:

(2)

where *w*_*i *_is the distance between the two lines in the pair, i.e. the expected shaft width. If grey-scale information is taken into consideration, as suggested in [15], it is intuitive that the intensity gradient of the leftmost line should change from dark to bright, and vice versa for the rightmost line. The mean of the magnitude of these gradients for each line should also be large, to detect a true bone contour. Therefore, the probability *p*_*i *_that a pair of lines *i *forms a shaft contour, based on both width between lines and intensity gradient, is given by:

(3)

where *M*_*i *_is the mean of the intensity gradient magnitudes of the points along both lines in the pair. Each candidate pair *i *has a probability *p*_*i *_of representing the femoral shaft, and the top four mostly likely candidate pairs are kept. To ensure that the correct two pairs are chosen, we use the distance between the left and right femur, measured from the inner contour of each. Again, the shafts were paired together, and the distribution was modelled as a Gaussian with *μ*_*d *_= 700.86 pixels and *σ*_*d *_= 34.53 pixels. The probability *p*_*j *_that a pair of detected shafts *j *accurately match the shafts in the actual image is given by:

(4)

where *d*_*j *_is the distance between the inner contours of the left and right femoral shafts. The shaft pair with the highest value of *p*_*j *_represents the final detected shaft position.

### Detection of femoral heads

**Figure 3:**
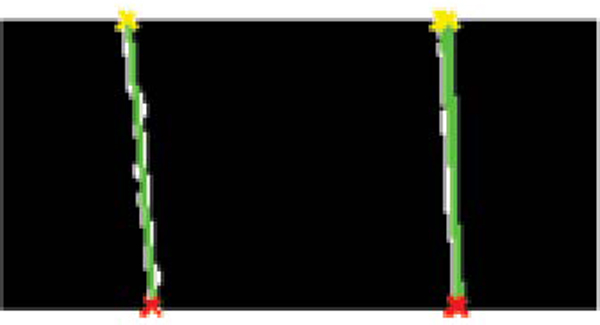
**Hough line detection of femoral shafts**. Results of directed Hough Transform for detection of femoral shafts.

**Figure 4:**
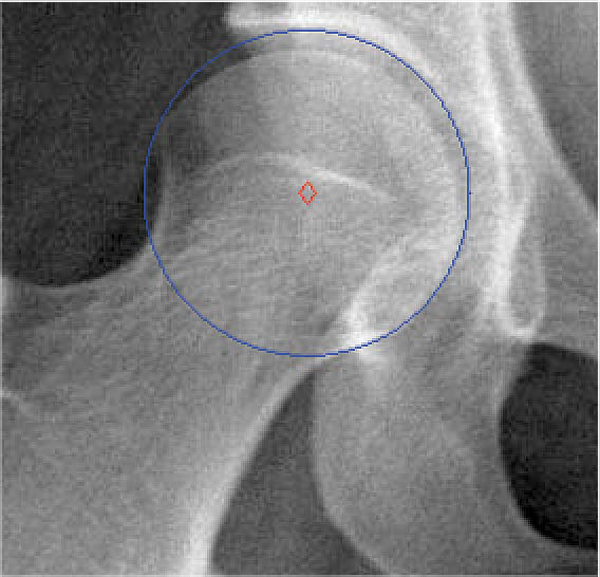
**Hough circle detection of femoral heads**. Results of directed Hough Transform for detection of femoral heads.

Once the positions of the femoral shafts are known, the patient's approximate horizontal displacement can be calculated as the horizontal distance between the center point of the shafts and the center of the image. This information is later used in detecting the pelvic ring, to aid in accurate initialization of the active shape model. However, the femoral shafts alone do not provide adequate knowledge of the patient's approximate vertical position. Therefore, the next step is to determine the location of the femoral heads; vertically, these are positioned at the base of the pelvic ring. Since the femoral heads are typically circular, directed Hough transform is again a suitable method. The parametric equation for a circle is:

(5)

where *r *is the circle radius, and *a *and *b *are the coordinates of the circle center. Hough transform for circle detection requires a 3D accumulator, increasing the computational complexity of the process. To both improve speed and reduce the probability that incorrect circles are found, we again restrict the area of search and also determine the probability of a circle being correct based on the width of the femoral shaft. The area of search is restricted to 200 pixels right of the left femur, and 200 pixels left of the right femur. Study of the dataset confirmed that the femoral heads typically appear in this area of the image. Vertically, the search is restricted to the bottom 2/3 of the image; even in training x-rays with severe vertical displacement, the femoral head is still visible within this portion. To prevent detection of undesired circular structures - such as circles formed by the outer curves of the pelvic structure - we restrict the circle radius to a range obtained from analysis of the full dataset (60-90 pixels). This avoids the majority of incorrect circles. With a larger dataset, we hope to construct another Gaussian distribution to estimate the probability of a detected circle representing the femoral head; however, using a pixel range did not affect the quality of our results. Figures 3 and 4 demonstrate the results of directed Hough Transform.

The approximate positions of the femoral heads are now known, and used to initialize the active shape algorithm for detection of the combined femur structure.

### General active shape model algorithm

In ASM, the target shape is defined by a set of landmark points. The first stage is to determine and label the landmarks in a set of training images, which will be used to generate the Point Distribution Model (PDM) that describes the target shape. The final labeled training set, *S*, contains *N *shapes with *k *landmarks each. Each shape is therefore represented as a vector of (*x*, *y*) coordinates defining each of its landmarks in sequence. Due to the wide variation across patients in pose angle and position, it is vital that the training shapes be correctly aligned. Given two shape vectors *z*_*i *_and *z*_*j*_, a transform  is calculated which aligns *z*_*i *_to *z*_*j*_, where *s *is the scaling value, *θ *is the rotation angle, *X*_*t *_is the horizontal translation and *Y*_*t *_is the vertical translation. These last two values are particularly vital in dealing with pelvic x-ray images, as there is no standard initial position for each patient. Note that these measures are automatically calculated by the algorithm. After alignment, PCA is applied, so each training shape can be approximated by:

(6)

where *P *contains the *t *eigenvectors of the covariance matrix, and *b *is a *t*-dimensional vector which defines the parameters for the deformable model. A limit is placed on the possible change in *b*, to ensure that the generated shape is similar to the shapes in the training set.

The training set is also used to build a statistical grey level model, by sampling the neighboring pixels of each landmark point. Specifically, a model is built for each separate landmark, by sampling the derivative of the intensity values along the profile normal to the landmark in each training image. These samples are normalized, and the mean  and covariance *S*_*g *_are evaluated for each landmark point. During each iteration of the shape matching process, a specific number of pixels are sampled along the profile normal to each current model point. The quality of fit for each is calculated as the Malanobis distance of the sample from the model mean, given by:

(7)

The pixel with the lowest value of *f*(*g*_*s*_) is the new "best" position for the model point; this is repeated for each point. The shape parameters *b *are then updated to fit these new positions. The algorithm halts when an iteration results in no significant changes in *b*.

### ASM for femur, pelvic ring and pubis detection

**Figure 5:**
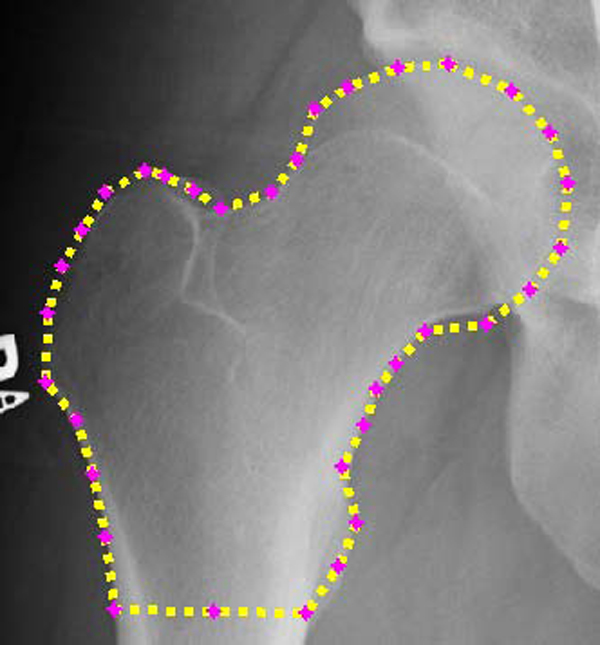
**Example ASM detection of left femur**. Example of left femur detection using ASM algorithm.

In this study, ASM is applied separately to detection of both the femurs and the pelvic ring. As with the femoral shafts, the pelvic ring is distinctly visible within the majority of x-rays, and suffers relatively little deformation in typical pelvic fractures. Determining its location in turn determines the approximate position of the iliac crests. However, the ASM algorithm is highly sensitive to initialization, and a poor starting position for the mean shape produces unsatisfactory results. Our system circumvents this via its hierarchical approach. The positions of the femoral shafts and heads are already known, following the use of directed Hough Transform. This knowledge is used to initialize the ASM femur model. During training, 35 landmarks are used to describe the femur; this choice was based upon experimental results. We limit the change in shape parameters to 4*σ*, where *σ *is the standard deviation; this allows the starting shape to deform to fit femur shapes at various angles without losing its base structure. In each iteration, the algorithm examines 20 pixels along the profile either side of the model point to find a new best position. Figure 5 illustrates an example of left femur detection using ASM.

Identifying the femurs in turn allows for correct initialization of the ASM shape for the pelvic ring; across all training images, it was found that the femoral heads and pelvic ring are typically *<*100 pixels apart horizontally. The uppermost landmark on the ASM model for each femoral head was selected as a ring reference point, and a horizontal search for the largest intensity gradient value was perfomed across a +100 pixel range from this point. A sudden change in grayscale intensity corresponds to the inside edge of the pelvic ring; the pixel with the largest value is recorded as a pelvic ring initialization point. After this is repeated for both femurs, the two initialization points are used in initializing the ASM shape for pelvic ring detection. Note that prior detection of the femurs is vital; the pelvic ring alone cannot be used as a reference structure for segmentation, as its horizontal and vertical position in the x-ray image is unknown. In the training phase, 30 landmarks are used to describe the pelvic ring; as with the femur model shape, these are a mixture of structural and auxiliary points. In this case, only 10 pixels either side of the profile point are searched, to prevent the shape from aligning to the internal gaps in the pubic bones beneath the pelvic ring. Shape parameter change is again limited to 4*σ*.

The ASM detected position of the pelvic ring is then used to initialize ASM for detection of the left and right pubis, via the use of control points along the base of the ring. In this case, 23 landmark points describe the outer edge of the pubis bone, and 8 landmarks describe the inner edge. Shape parameter change is limited to 3*σ *and 15 pixels are searched either side of each profile point.

Since speed is important in a medical trauma environment, our approach employs a multi-resolution version of the ASM algorithm. Each training and test image is decomposed into multiple lower resolution images, each a smoothed and subsampled version of the last; these correspond to levels of a Gaussian pyramid. The search for the desired shape begins at the highest level: the lowest resolution image. When the ASM algorithm converges at a specific level - i.e. there is no significant change in the model points - the next highest resolution image is used. In this way, the location of the identified shape is refined over progessively higher resolutions. The original image is considered only at the final stage of the process, when only slight changes are expected in the model point positions. The algorithm in this paper uses 3 levels for detection of the pelvic ring, as its simple shape remains intact at the lowest image resolution. When using ASM to detect the femurs and pubis, it was found that the model shapes suffered considerable distortion at low resolutions, and therefore 2 levels were used. For detection of the iliac crests, only one level was used.

### Detection of iliac crests

The left and right iliac crests are typically circular, and directed Hough transform can therefore be used to detect their approximate location. However, depending on the patient pose angle, one crest may appear hooked, rather than circular. Analysis of the dataset revealed that at least one the crests was always detected. This is intuitive from knowledge of pelvic structure; within the expected range of patient rotation angles, at no point would both crests become distorted. Analysis also supported the assumption of approximate symmetry about the pelvic ring. Therefore, the position of the detected crest can be used to determine the position of the undetected crest. Once directed Hough transform has determined the approximate location of an iliac crest, the ASM starting shape is initialized using a specific reference point on the Hough transform circle, as well as knowledge of the pelvic ring position. The iliac crest shape is described by 22 points, shape parameter change is limited to 5*σ*, and 35 pixels are searched either side of each profile point. As with all selected ASM parameters, these values were determined empirically.

## Results and discussion

### Evaluation

#### ANOVA test

Since the purpose of the hierarchical algorithm described in this paper is to automatically initialization detection of key pelvic structures, performance is compared versus manually initialized ASM. This second set of examples is generated by a user manually placing the ASM template within the desired structure in the x-ray image, and then running the ASM algorithm. Since our algorithm is designed for hierarchical automated initialization of ASM, our primary concern is how it performs versus manual initialization, rather than how ASM itself performs on the pelvic structures. Note that in several cases, ASM is insufficient to correctly detect structures regardless of initialization; we are further developing a form of ASM that maintains shape curvature and offers greater control over deformation.

Across a set of 20 images, three key structures - the left iliac crest, left femur and pelvic ring - are manually labeled and taken as the 'true' reference shapes. For each image, the difference in area between this reference shape and the shape detected via our algorithm is calculated and normalized by the area of the reference shape. In other words, where *A *is the reference shape (i.e. a set of pixels) and *B *is the shape detected via our algorithm, our normalized error measure *e *is calculated as:

(8)

The same error measure is calculated for the reference shape and the shape detected via manually-initialized ASM. ANOVA is then performed for all three structures to determine whether there is a significant difference between results obtained via manual initialization and results obtained using our hierarchical automatic initialization algorithm.

**Figure 6:**
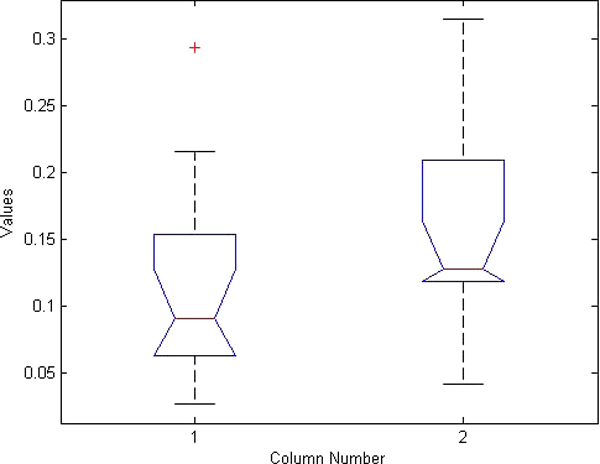
**ANOVA boxplot for left iliac crest detection**. Boxplot generated from ANOVA test for left femur detection results using manual ASM initialization and using our hierarchical automated algorithm.

**Figure 7:**
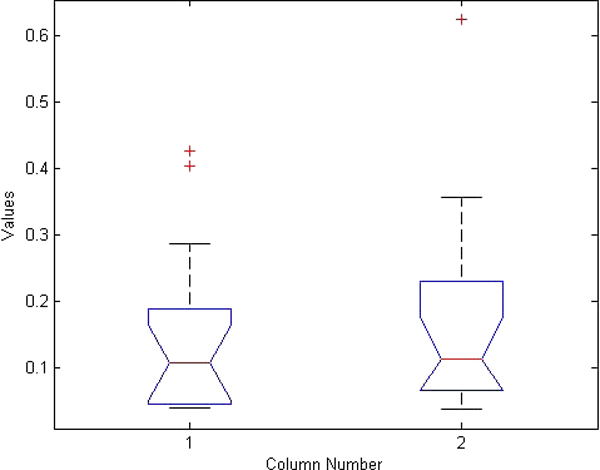
**ANOVA boxplot for left femur detection**. Boxplot generated from ANOVA test for left iliac crest detection results using manual ASM initialization and using our hierarchical automated algorithm.

**Figure 8:**
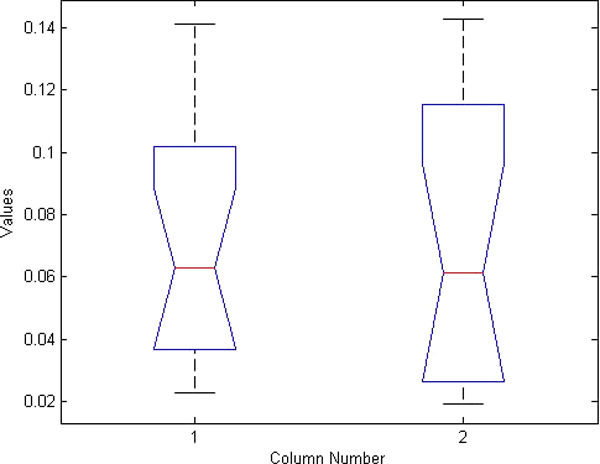
**ANOVA boxplot for pelvic ring detection**. Boxplot generated from ANOVA test for pelvic ring detection results using manual ASM initialization and using our hierarchical automated algorithm.

When comparing results for pelvic ring detection, ANOVA calculates a p-value of 0.8431. For iliac crest detection, *p *= 0.0776, and for femur detection *p *= 0.6078. These p-values indicate no significant difference between results for manual initialization and our automatic method. It should be noted that in all three cases, the sum of normalized error measure for our automated method is less than the sum for manual initialization. This is particularly noticable in iliac crest detection, explaining the lower p-value for that ANOVA test. Figures 6, 7 and 8 present the ANOVA boxplots for left iliac crest detection, left femur detection and pelvic ring detection respectively. It can be seen that the automatic initialization offers slightly more consistent results, with less variance.

#### Visual inspection

As with many image-processing techniques, visual inspection may be helpful in evaluating performance. The results were evaluated by two radiologists and one trauma surgeon. Detection of each structure was classified across the fifteen test images into three categories: Good, Acceptable, and Unacceptable. These categories are subjective, but since our algorithm is trained on a set of human-labeled images, the classification provides useful feedback. The results are presented in Table 1. The left and right pubis show a number of unacceptable results. However, the crests, femur and pelvic ring are almost always detected to at least an acceptable standard; typically, segmentation is rated as good.

**Table 1:** Visual inspection results

**Structure**	**Good**	**Acceptable**	**Unacceptable**
Left Iliac Crest	11	4	0

Right Iliac	12	2	1

Pelvic Ring	10	5	0

Left Pubis	9	1	5

Right Pubis	10	1	4

Left Femur	12	3	0

Right Femur	11	3	1

Segmentation performance of all key structures over 15 test images, as evaluated by experts.

**Figure 9:**
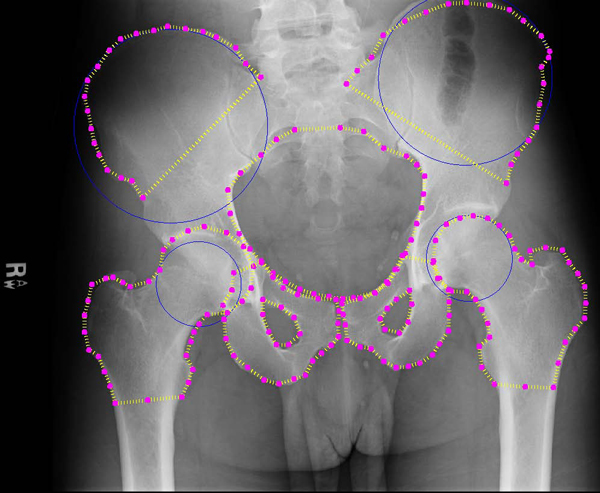
**Automated detection results: first example**. Example results for automated detection of all key pelvic structures. Results are accurate for all regions.

**Figure 10:**
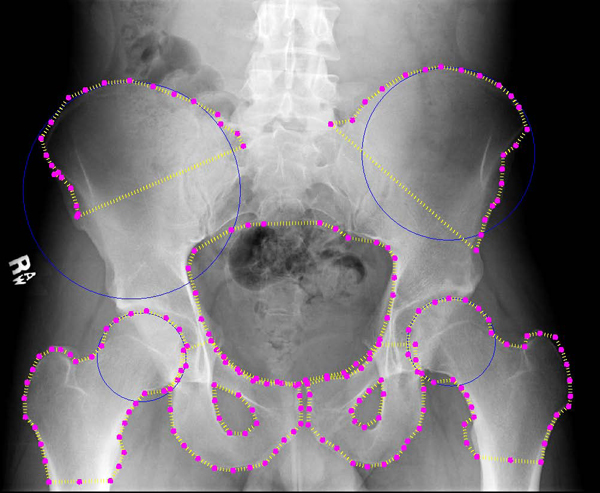
**Automated detection results: second example**. Example results for automated detection of all key pelvic structures. Results are accurate for all regions.

Figures 9 and 10 illustrate some successful segmentation examples. Edge detection is generally accurate for all key structures. This suggests that the automated algorithm performs accurate initialization of the ASM starting shape. In both Figures 9 and 10 the detected right iliac crest is not smooth, but this is expected to improve by applying prior image processing to enhance the dark outer edges of the crest. The crucial outcome is that the algorithm locates the correct region for each structure and therefore successfully initializes ASM, with performance at least equal to manual initilization by the user. These areas can later be analyzed to detect particular types of fracture. The hierarchical nature of our method allows for rapid and accurate detection of these areas, using basic anatomical information and knowledge gained at each step of the process.

**Figure 11:**
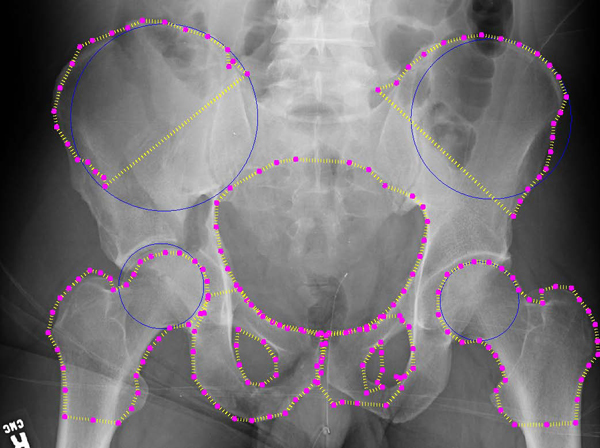
**Automated detection results: third example**. Example results for automated detection of all key pelvic structures. Results are good for pelvic ring, right crest and left pubis. Performance on other structures demonstrates some issues.

**Figure 12:**
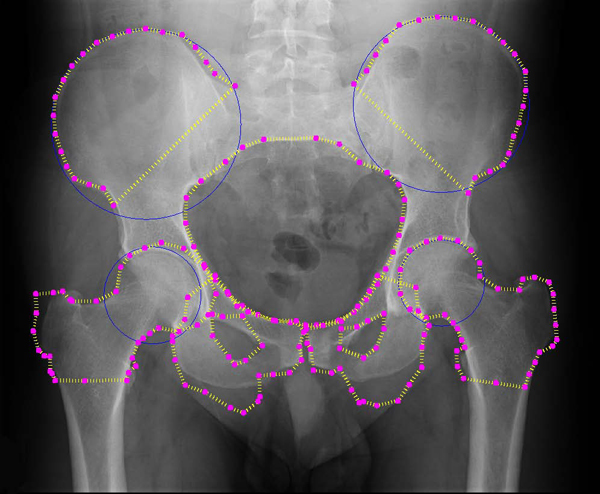
**Automated detection results: fourth example**. Example results for automated detection of all key pelvic structures. Results are good for pelvic ring, both crests and the right femur. Performance on other structures demonstrates some issues, particularly left and right pubis.

Figures 11 and 12 demonstrate some difficulties in segmentation. In Figure 11, detection of some structures is very accurate (left femur, right iliac crest, pelvic ring, left pubis), but detection of the right pubis is distorted, and part of the left iliac crest is excluded. The first issue may be due to the variation in pubis bone shape and angle across the dataset and may be improved by training across a wider range of patients or using more control points during initialization. Detection of the left crest, meanwhile, appears to have converged towards an internal 'false edge' with similar greyscale intensity statistics to the true edge. Figure 12 demonstrates excellent detection of the crests and ring and reasonable detection of the femurs.

Detection of the pubis bones, however, is less accurate; this may be due to the reason described above.

## Discussion

The results of our algorithm on test images are promising, and the hierarchical approach greatly aids in correct initialization of the ASM shape algorithm.

Despite occasionally poor detecton of contours, it can be observed in all images that the general position of each structures was correctly determined. This will allow each area to be analyzed for specific fracture characteristics. Furthermore, the similarity in results between our automatic algorithm and manual initialization suggests that the detection issues are due to ASM itself. Independently of this, ANOVA results indicate that our automatic initialization algorithm performs at least as well as manual initialization in detection and segmentation of key pelvic structures.

As evaluated by two radiologists and one trauma surgeon, detection of the crests, the ring, and the femurs was generally accurate in almost all cases. Detection of the pubis proved more of a challenge, but we intend to use more control points to control placement and perhaps prior deformation of the template shape before performing ASM detection. In some cases, artifacts on the x-rays caused the detection of false edges; if these can be distinguished from bone, possibly via texture analysis, these false edges can be avoided. The algorithm performs fairly well in the presence of background intensity variations. In Figure 9 it can be seen that the detection of the iliac crests was successful despite the dark edges of the structure compared to other example images. This indicates that the preprocessing of the images was successful in alleviating brightness and constrast variations. Background soft tissue, which can display similar intensity statistics to bone, was also correctly excluded from the segmentation results. Though there is frequent noise and intensity variation within the pelvic ring area, this structure was also successfully detected. Improvements can be made to the algorithm to compensate for the significant histogram differences between images. This preprocessing would naturally be automatic, to be integrated fully into the existing system.

Our method is fully automated, and, excluding the need for labelled training examples, no input is required from the user. The number of training examples required is also relatively low. As our dataset consists of pelvic injury patients, we have observed that the algorithm performs well in the presence of fracture. By isolating specific pelvic regions, we can then search these areas for fracture via such methods as texture analysis. Exceptions will occur when the pelvic ring is completely disrupted or the femurs are severely fractured. However, these injuries are so severe and so clearly visible that it is unlikely our system would be necessary.

Fractures of the pubis may be challenging to detect using our method in its current form, as the superior ramus and inferior ramus can break into two separate parts. This creates a gap in the bone edge. However, ASM may actually aid in detecting such fractures. if we are able to superimpose the general shape on the x-ray, we will be able to detect sudden dark spots (i.e. gaps) along the bone edge. The success of this approach will depend on alterations made to the fitness function, to better preserve the shape of the pubis in the presence of significant edge disruption.

Time complexity for the algorithm is acceptable; when tested on an Intel quad-core machine with 4 GB RAM, running time for a single image was approximately 1 minute. As the most time-consuming step is Hough circle detection, it is likely that the running time can be decreased by reducing the range of radii searched. This can be achieved by modelling the ratio of femoral head radius to femoral shaft width as a Gaussian, as outlined in [15], and searching the 95th percentile of the normal cove. We believe the same approach will apply to Hough detection of the iliac crests; preliminary analysis of the dataset suggests a relationship between crest radius and pelvic ring width. The ASM steps were quickly completed, which is likely due to the multi-resolution approach.

Although training is required for the algorithm to perform successfully, based on the current approach we anticipate it would need to be performed once per x-ray machine, in order to take account of differences in grey-scale range and intensity. Note that once these differences are equalized via preprocessing - an area we are currently exploring - training could be done offsite, using a separate database of past patients, prior to deployment of the system.

## Conclusion

This paper provides a automated hierarchical method for segmentation of key structures from pelvic x-ray images: the iliac crests, the femurs, the pelvic ring and the pubis bones. First, directed Hough transform is used to detect the femur shafts, and so correctly determine the patient's horizontal position within the image. Hough transform is then combined with ASM to detect the femur - and in turn, the acetabulum. The position of the femurs is used to initialize ASM for pelvic ring detection, and the location of the pelvic ring is then used to initialize ASM for the pubis bones and to direct Hough transform for detection of the iliac crests. After the general position of the crests is known, ASM is applied. Our method offers several improvements over existing approaches. First, and most crucially, it is entirely automatic, requiring no user input other than specifying the patient's gender. It also performs accurately in the presence of fracture and deals with several issues affecting x-ray imaging in a clinical environment; specifically, uncertain pose angle and position of patient, and greyscale variations caused by differences between x-ray machines.

Future work will focus on refinement of the algorithm, particularly in dealing with a wider range of patient pose angles. As future work, we will also incorporate the Hough transform results into the active shape model algorithm for weighting, as well as for initialization of the starting shape. The Active Appearance Model algorithm (AAM) will also be tested for comparision; this has also been used within the literature for segmentation of medical images. We also plan to more finely control the deformation of the ASM template via the use of splines for maintaining curvature. After the segmentation process has been refined, work will begin on fracture detection within the identified structures. This will be integrated into a more comprehensive system under development, where details of fracture type and severity can be combined with physiological and demographic information to provide accurate diagnostic recommendation to physicians.

## Competing interests

The authors declare that they have no competing interests.

## Authors' contributions

RS and KN have designed the algorithms, analyzed the data, and drafted the manuscript. KW provided expert medical knowledge and assisted in evaluation of results. All authors have equal participation in the study as well as preparation of the final paper.

## References

[B1] Basta A, Blackmore C, Wessells H (2007). Predicting urethral injury from pelvic fracture patterns in male patients with blunt trauma. J Urology.

[B2] Schmal H, Markmiller M, Mehlhorn A, Sudkamp N (2005). Epidemiology and outcome of complex pelvic injury. Acta Orthopaedica Belgica.

[B3] Tan S, Porter K (2006). Free fall trauma. J Trauma.

[B4] ACOS (2004). ATLS Advanced Trauma Life Support Program for Doctors.

[B5] Haas B, Coradi T, Scholz M, Kunz P, Huber M, Oppitz U, Andre L, Lengkeek V, Huyskens D, van Esch A, Reddick R (2008). Automatic segmentation of thoracic and pelvic CT images for radiotherapy planning using implicit anatomic knowledge and organ-specific segmentation strategies. Phys Med Biol.

[B6] Pasquier D, Lacornerie T, Vermandel M, Rousseau J, Lartigau E, Betrouni N (2007). Automatic segmentation of pelvic structures from magnetic resonance images for prostate cancer radiotherapy. Int J Radiat Oncol Biol Phys.

[B7] Kane SA (2002). Introduction to Physics in Modern Medicine.

[B8] Ding F, Leow WK, Howe TS (2007). Automatic segmentation of femur bones in anterior-posterior pelvis x-ray images. Computer Analysis of Images and Patterns.

[B9] Begg JD (2006). Abdominal X-Rays Made Easy.

[B10] Manos G, Cairns A, Rickets I, Sinclair D (1993). Segmenting radiographs of the hand and wrist. Computer Methods and Programs in Biomedicine.

[B11] Vittitoe N (1998). Identification of lung regions in chest radiographs using Markov random field modeling. Medical Physics.

[B12] Duryea J, Boone J (1995). A fully automated algorithm for the segmentation of lung fields on digital chest radiographic images. Medical Phys.

[B13] Pietka E (1994). Lung segmentation in digital radiographs. Journal of Digital Imaging.

[B14] McNitt-Gray M, Huang H, Sayre J (1995). Feature selection in the pattern classification problem of digital chest radiograph segmentation. IEEE Trans on Medical Imaging.

[B15] Chen Y, Ee X, Leow WK, Howe TS (2005). Automatic extraction of femur contours from hip x-ray images. Computer Vision for Biomedical Image Applications.

[B16] MG R, Cootes T, Adams J (2006). Vertebral morphometry: semiautomatic determination of detailed shape from dual-energy X-ray absorptiometry images using active appearance models. Invest Radiol.

[B17] Behiels G, Vandermeulen D, Maes F, Suetens P, Dewaele P (1999). Active Shape Model-Based Segmentation of Digital X-ray Images. MICCAI '99: Proceedings of the Second International Conference on Medical Image Computing and Computer-Assisted Intervention.

[B18] Boukala N, Favier E, Laget B, Radeva P Active shape model based segmentation of bone structures in hip radiographs. Industrial Technology, 2004 IEEE ICIT '04 2004 IEEE International Conference on 2004.

